# The Underscreening of Cardiac Amyloidosis in Patients With Pacemakers—A Single Centre Retrospective Audit

**DOI:** 10.1111/jce.70323

**Published:** 2026-03-19

**Authors:** Peishan Cai, Timothy Scully, Jason Nogic, Kah Peck, Jithin Sajeev

**Affiliations:** ^1^ School of Medicine Deakin University Geelong Victoria Australia; ^2^ Department of Cardiology Box Hill Hospital Box Hill Victoria Australia; ^3^ Eastern Health Clinical School Monash University Melbourne Victoria Australia

**Keywords:** conduction system disease, heart failure, pacemaker, screening, transthyretin cardiac amyloidosis

## Abstract

**Background:**

Cardiac amyloidosis is commonly associated with cardiac conduction disease. We sought to determine the prevalence of advanced conduction disease requiring a pacemaker in patients with known cardiac amyloidosis to evaluate current screening practices among patients receiving pacemakers.

**Methods:**

A two‐stage analysis was conducted. First, a retrospective chart review was conducted for all patients with known transthyretin cardiac amyloidosis who attended the Cardiac Amyloidosis clinic at the Victorian and Tasmanian Amyloidosis Service (Australia) from 2014 to 2024 to identify those with a pacemaker implantation. Second, all patients who received a pacemaker at the same centre, were reviewed for the presence of cardiac amyloidosis. Demographic and clinical information was collected to identify “red flags” suggestive of cardiac amyloidosis and the patients that underwent screening were identified.

**Results:**

Of those patients with known transthyretin cardiac amyloidosis, 82 (39.8%) patients required a pacemaker for conduction disease. Of the 470 patients who had a pacemaker inserted and did not have a pre‐existing diagnosis of cardiac amyloidosis, 50 (10.6%) patients had at least 3 red flags for cardiac amyloidosis. However, only five patients (1.1%) were screened for cardiac amyloidosis and four (80% of those screened) were diagnosed with the condition.

**Conclusions:**

A significant proportion of patients with transthyretin cardiac amyloidosis had pre‐existing pacemakers. However, only a small proportion of patients who required a pacemaker underwent screening, despite the presence of multiple high‐risk features.

## 1 Introduction

Transthyretin cardiac amyloidosis (ATTR‐CA) is an infiltrative cardiomyopathy caused by the deposition of misfolded transthyretin protein into the myocardium [1]. With the advent of disease modifying treatment options, early detection has become increasingly important. However, the optimal method of screening for ATTR‐CA remains unclear. Currently, patients with heart failure (HF) represent the primary group screened for ATTR‐CA [2]. However, this population tends to be older and have more advanced disease [2]. Current treatment of ATTR‐CA primarily consists of transthyretin stabilisers or silencers, which slow rather than reverse disease progression, therefore, earlier detection and initiation of treatment may confer clinical benefit [1].

Screening for ATTR‐CA also involves screening for light chain cardiac amyloidosis (AL‐CA), another infiltrative cardiomyopathy [3]. As both conditions have different management and prognosis, differentiation at diagnosis is crucial [3]. Cardiac conduction disease may be a manifestation of ATTR‐CA and AL‐CA.

We conducted this study with two aims: first, to determine the prevalence of severe conduction disease requiring pacemakers among patients with known ATTR‐CA; second, to evaluate current screening practices of both ATTR‐CA and AL‐CA in patients requiring a pacemaker.

## 2 Methods

A single centre retrospective analysis using the hospital's electronic medical records was performed on two patient populations. Demographic and clinical information was collected for both patient populations. The first group consisted of patients with known ATTR‐CA, who were identified using a database created from records of patients who accessed the centre's cardiac amyloidosis clinic, a statewide amyloid service. The second group included all patients that received a pacemaker between April 2014 and June 2022. Information collected on chart review of the group that received a pacemaker included identification of red flags at time of pacemaker insertion, and details on screening and diagnosis of cardiac amyloidosis. Red flags were defined as male sex, history of carpal tunnel syndrome (CTS) or spinal laminectomy, atrial fibrillation (AF), increased left ventricular wall thickness (≥ 12 mm), low voltage ECG (< 5 mm in all limb leads) and evidence of plasma cell dyscrasia. Diagnosis of CTS/spinal laminectomy was based on clinical notes. Diagnosis of AF was assessed through clinical notes and baseline rhythm recorded during investigations and procedures. Evidence of plasma cell dyscrasia was based on pathology and clinical documentation. Screening consisted of a serum protein electrophoresis and bone scintigraphy. A positive diagnosis of ATTR‐CA requires a normal serum and urine protein electrophoresis, immunofixation and a Perugini stage 2 or 3 on bone scintigraphy [1]. Suspicion of AL‐CA occurred when there is an abnormal serum protein electrophoresis irrespective of bone scan results, and this would prompt further workup [4].

## 3 Results

Of the 206 patients with known ATTR‐CA, 82 patients (39.8%) had pacemakers. 51.7% of patients had their pacemaker inserted prior to ATTR‐CA diagnosis. The earliest pacemaker insertion was 18 years prior to ATTR‐CA diagnosis whilst the latest was 6 years post diagnosis. Where information was available, the most common reason for pacemaker insertion for ATTR‐CA patients was atrioventricular (AV) nodal disease (75%), sinoatrial nodal disease (15.9%) or as part of a pace and ablate strategy (9.1%). Most ATTR‐CA patients received a dual chamber pacemaker (61.5%), followed by single chamber devices (23.1%) and biventricular devices (15.4%).

The second part of our study comprised of 470 patients who received pacemakers. Median follow‐up period was 60 months (IQR: 36–96). Median age at device implantation was 81 (interquartile range [IQR]: 76–86) years. Two hundred and fifty‐six patients (54.5%) were male. Two hundred patients (42.6%) had at least two red flags for cardiac amyloidosis in addition to conduction disease and 50 had at least three red flags (10.6%) (Table 1). Five patients were screened for cardiac amyloid. Three patients had ATTR‐CA and one had AL‐CA. Diagnosed patients all had at least three additional red flags. All patients were screened primarily due to HF symptoms. Two patients also had echocardiographic findings suggestive of cardiac amyloidosis. One patient had left ventricular wall thickening whilst another also had decreased longitudinal strain and apical sparing. Two patients had New York Heart Association (NYHA) class II symptoms whilst three had NYHA class III symptoms.

**Table 1:** Analysis of pacemaker population for red flags, screening and diagnosis of transthyretin cardiac amyloidosis.

**Characteristics of participants**	All patients (*n* = 470)
Median Follow‐up duration post pacemaker (months)	60 (36–96)
Median pacemaker insertion age	81 (76–86)
**Screening for cardiac amyloidosis following pacemaker insertion**	
Number of patients screened for cardiac amyloidosis	5 (1.1%)
Number of patients diagnosed	4 (0.9%)
**Red flags for cardiac amyloidosis** d	
Male	256 (54.5%)
Atrial fibrillation	201 (42.7%)
Carpal tunnel syndrome or spinal laminectomy	31 (6.7%)
Low voltage criteria on ECG^a^	30 (6.4%)
Increased LV wall thickness (≥ 12 mm)^b^	125 (28.5%)
Plasma cell dyscrasia^c^	13 (2.8%)
**Red flag distribution**	
Number of patients with zero red flags	90 (19.2)
Number of patients with one red flags	188 (40.0%)
Number of patients with two red flags	142 (30.2%)
Number of patients with three red flags	43 (9.1%)
Number of patients with four red flags	7 (1.5%)
Number of patients with five red flags	0 (0.0%)

^a^ 27 patients excluded from calculation due to missing data.

^b^ 155 patients excluded from calculation due to missing data.

^c^ Plasma cell dyscrasia bloodwork is not routinely performed in absence of clinical indication, therefore patients without appropriate bloodwork are presumed to not have plasma cell dyscrasia.

d Percentage of red flags calculated using the total number of patients checked for the relevant red flags.

Analysis of red flags in patients who were and were not screened found that the risk factors of male, left ventricular wall thickening, plasma cell dyscrasia and a low voltage electrocardiograph were associated with an increased likelihood of patients being screened for cardiac amyloid (Table 2).

**Table 2:** Comparison of patients who were and were not screened for cardiac amyloidosis.

	Screened (*n* = 5)	Not screened (*n* = 465)	*p*‐value
Sex (male)	5 (100.0%)	251 (54.0%)	0.04*
Atrial fibrillation	3 (60.0%)	198 (42.6%)	0.43
Carpal tunnel syndrome/spinal laminectomy	1 (20.0%)	30 (6.5%)	0.23
Left ventricular wall thickening	5 (100.0%)	120 (27.6%)	< 0.001*
Plasma cell dyscrasia	1 (20.0%)	15 (3.2%)	0.04*
Low voltage ECG	2 (40.0%)	28 (6.0%)	0.002*

*Note:* Asterisk (*) denotes statistically significant values < 0.05.

## 4 Discussion

Our study found that the prevalence of pacemakers in patients with ATTR‐CA was 39.4%. 51.7% of these patients had a pacemaker inserted prior to ATTR‐CA diagnosis. Therefore, 20.4% of ATTR‐CA patients in our cohort had a pacemaker at time of diagnosis, which is similar to the 15% found in previous studies [2]. Our study found that the proportion of pacemakers inserted for AV nodal disease was greater in ATTR‐CA patients compared to the general population [5]. Seventy‐five percent of patients in our study required a pacemaker due to AV nodal disease, compared to 33% of the general population in 2009 [5].

The present study may help identify a high‐risk patient cohort suitable for cardiac amyloidosis screening. In our study only 1.1% of patients who received pacemakers were screened despite 9.1% having at least three red flags in addition to a pacing requirement. This indicates that the potential diagnosis of cardiac amyloidosis is often not considered in patients with a pacing requirement. However, these red flags should only function as an exploratory framework and not a validated screening tool since many of the listed red flags such as AF and male are highly prevalent in this population. Additionally, despite CTS being associated with cardiac amyloidosis, univariate analysis demonstrated that it did not prompt screening.

A study by Cannie et al. analysing participants aged between 70 and 85 years old found that 7.7% of patients with a pacemaker and high degree AV block had undiagnosed ATTR‐CA [6]. Another study analysing patients above 65 years old with a pacemaker and left ventricular wall thickening found ATTR‐CA in 19% of patients screened [7]. Therefore, patients with conduction disease requiring pacemakers may be a suitable screening population. Further research with a larger sample size is required to validate this hypothesis. Inclusion of other recognised high‐risk features for ATTR‐CA may further improve the diagnostic yield in this cohort.

Screening for ATTR‐CA within the appropriate time window is important. Screening too early may yield a false negative result, while screening too late may limit the efficacy of current therapies [1, 8]. Previous studies have found that up to 16% of patients with ATTR‐CA who were referred for screening due to HF exacerbations had a pacemaker implanted prior to ATTR‐CA diagnosis [2]. This suggests that pacemaker implantation in ATTR‐CA patients may pre‐date significant HF progression in some patients and therefore represent a favourable time window for treatment initiation. Hence, conduction disease requiring intervention may arise at a favourable time window for screening. In conclusion, pacemaker implantation is common in patients with ATTR‐CA, yet screening for cardiac amyloidosis remains limited in those with conduction disease requiring pacing. Given the availability of disease‐modifying therapy, prospective trials should identify risk factors and optimal screening strategies for early detection. As there are currently no screening scores for ATTR‐CA in pacemaker patients, utilisation of more general ATTR‐CA screening scores such as the ATTR‐CM score and T‐Amylo score may facilitate early diagnosis [9, 10].

## Funding

The authors received no specific funding for this work.

## Ethics Statement

Eastern Health Office of Research and Ethics has granted ethics for this study as a part of a larger trial (HREC/106104/EH‐2024‐427655).

## Conflicts of Interest

The authors declare no conflicts of interest.

## Data Availability

Data are available upon reasonable request.
